# Method to extract minimally damaged collagen fibrils from tendon

**DOI:** 10.14440/jbm.2016.121

**Published:** 2016-09-16

**Authors:** Yehe Liu, Nelly Andarawis-Puri, Steven J. Eppell

**Affiliations:** ^1^Department of Biomedical Engineering, Case Western Reserve University, Cleveland, OH 44106, USA; ^2^Leni and Peter W. May Department of Orthopaedics, Icahn School of Medicine at Mount Sinai, New York, NY 10029, USA

**Keywords:** extraction method, collagen, fibril, tendon

## Abstract

A new method is presented to extract collagen fibrils from mammalian tendon tissue. Mammalian tendons are treated with a trypsin-based extraction medium and gently separated with tweezers in an aqueous solution. Collagen fibrils released in the solution are imaged using both dark-field light microscopy and scanning electron microscopy. The method successfully extracts isolated fibrils from rat tail and patellar tendons. To examine whether the method is likely to damage fibrils during extraction, sea cucumber dermis fibril lengths are compared against those obtained using only distilled water. The two methods produce fibrils of similar lengths. This is contrasted with fibrils being shortened when extracted using a tissue homogenizer. Scanning electron microscopy shows the new method preserves D-banding features on fibril surfaces and that fibril diameter does not vary substantially compared with water extracted fibrils.

## INTRODUCTION

Many mammalian tissues are constructed primarily of collagen fibrils. Collagen fibrils self-assemble when triple helical collagen molecules form intermediate structures called microfibrils [1] which subsequently grow or aggregate into fibrils with diameters ranging from a few nanometers to a few hundred nanometers and lengths ranging from microns to millimeters [2,3]. Microscopic investigation of collagen fibrils dates back at least 60 years [4]. These microscopic studies have been used to explain properties of biological systems ranging from mineralization [5] to optical properties of the cornea [6]. Composed of more than 85% dry mass of collagen, tendon is the tissue with the highest collagen content. To study the properties of mammalian tendons, several groups, including our own, currently operate under the assumption that a first principles understanding of tissue physical properties requires knowledge of the mechanics of individual fibril elements and have begun to perform experimental and theoretical studies of fibrils [7-14]. Such studies are complicated by the presence of non-collagenous material in the test sample. The complications can be avoided by extracting single fibrils from tissues. This paper contributes a new method by which the collagen fibril extraction from tendon can be accomplished.

Isolated collagen fibrils, mostly from dermis tissue, have been studied extensively using various extraction methods [15,16]. However, methods to isolate individual collagen fibrils from tendon tissue without harsh treatment are lacking. In part, this is why our previous work on single fibrils was done using fibrils extracted from sea cucumber dermis [17-19]. This esoteric choice was made because the tissue was amenable to fibril extraction using simple dissection followed by swelling in water. However, there is an interest in moving away from dermis collagen fibril because collagen fibrils in different tissue can be structurally different, which leads to different structural functionality [20]. Such structure-function relationships are well studied; they can even occur within the same tissue under disease states [21].

Until recently, extraction of collagen fibrils from tendon tissue was only achieved using harsh treatments. In several related studies, single fibrils were extracted from rat [22,23], bovine [24,25] and human [26] tissues. Vortexing damaged 88% of extracted fibrils [22]. Mechanical spreading with tweezers followed by nitrogen drying worked well for measurements made under dry conditions [26], but drying fibrils irreversibly changes their properties [11,20]. Blending with a tissue homogenizer also effectively separates fibrils [24,25], but we show below that this results in broken and shortened fibrils. In summary, concerns exist that previously reported techniques for isolating fibrils from tendon might damage the fibrils yielding significantly different fibril morphology than would be found in native fibrils. We thus set out to find an alternative method allowing for tendon fibril extraction with less vigorous insult to the target tissue’s fibrils.

This paper presents a new enzymatic based method developed to extract fibrils that is less physically disruptive than homogenization-based methods. The primary goals of the paper are twofold. First, to show the new method yields enough collagen fibrils from tendon tissue to perform many single fibril tests. Second, by comparing with sea cucumber dermis fibrils extracted by simple extraction in water, to show the new method produces fibrils that are morphologically similar to water-extracted fibrils. The enzymatic method is shown capable of extracting fibrils from not only rat tendons, but also sea cucumber dermis. The method caused measurably less morphological damage to collagen fibrils compared with tissue homogenization as determined by quantitative measures of extracted fibril lengths.

**Figure 1.**
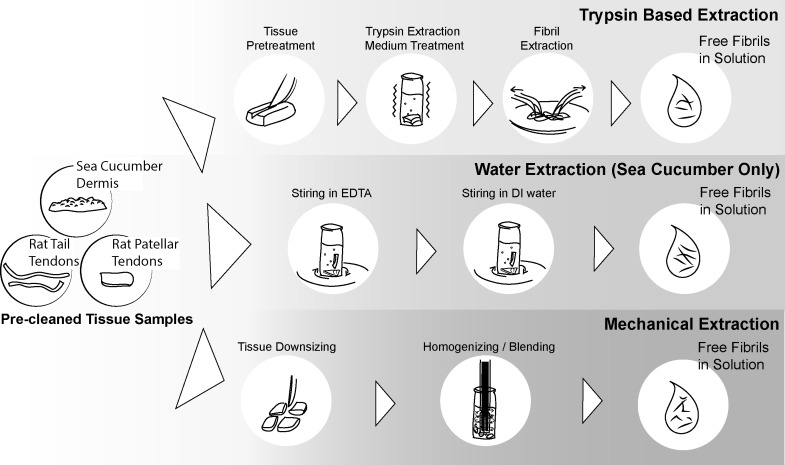
**Three collagen fibril extraction methods discussed in this paper.** The trypsin based extraction method, the new fibril extraction method introduced in this paper, is compared with two previously commonly used fibril extraction methods. Top: the trypsin base extraction method has 3 major steps: (1) disrupting the exterior sheath by cutting; (2) treating the tissue with trypsin; and (3) combing the swollen tissue to release free fibrils into the solution. Middle: the water extraction method has 2 major steps: (1) stirring the tissue in Tris-buffered EDTA, and (2) stirring the tissue in deionized water to release free fibrils into the solution. Top: the mechanical extraction has 2 major steps: (1) downsizing the tissue to optimized size; and (2) peforming vigrous mechanical disruption homogenization/blending/vortexing to release free fibrils into the solution

## MATERIALS AND METHODS

### General strategy

Three methods were used to extract fibrils from three different tissue sources: rat tail tendon, rat patellar tendon, and sea cucumber dermis (**Fig. 1**). The least aggressive method, “water extraction”, is based on a published method for extracting sea cucumber dermis fibrils [27]. However, this method is only effective on sea cucumber dermis. The most disruptive method, “mechanical extraction”, is based on standard blending and tissue homogenization techniques. The new method, “trypsin based extraction”, is slightly more chemically aggressive than water extraction, but less mechanically disruptive than tissue homogenization.

### Biological materials

Tail and patellar tendons were harvested from 8-week-old Lewis rats immediately following euthanization. The rats were drawn from the wild type control group of an unrelated study. The tissues were stored in phosphate buffered saline (PBS) and frozen at −80^°^C. Before each experiment, samples were thawed and stored at 4^°^C for < 72 h before the experiments. Rat tail tendon fascicles were harvested from unskinned rat tails using a previously published method [28]. Patellar tendons were dissected from rat hind-limbs after most of the non-collagenous tissues were removed from the surface. It was not possible to extract fascicles from the patellar tendons in a way analogous to that used in the tail. Optical microscopy and scanning electron microscopy of the patellar tendons showed no evidence of discrete fascicles existing in the rat patellar tendon (data not shown). Sea cucumber dermis was dissected from the marine species *Cucumaria frondosa*. Collagen monomer solution was 3 mg/ml lightly pepsin digested bovine skin collagen in 0.01N HCl, (Purecol, San Diego, CA, USA).

### Chemical materials

PBS was prepared by diluting 10 × PBS stock solution (Sigma-Aldrich, St. Louis, MO, USA) 1:9 in deionized (DI) water (MilliQ-UV Plus, Millipore, Billerica, MA) with pH adjusted to 7.4 using 1N hydrochloric acid. Fibril extraction involved a pre-treatment where tissues were swollen by shaking in a solution referred to as “extraction medium”. The extraction medium contained 4 mM ethylenediaminetetraacetic acid (EDTA), 0.1 M tris-HCl and 0.05% sodium azide at pH = 7.5–8.0 at 20^°^C [19]. Fibril extraction from tendon tissue required adding > 1000 BAEE Unit/ml type I trypsin from bovine pancreas (Sigma-Aldrich) referred to as “trypsin extraction medium”.

### Tissue pretreatment

At room temperature, all tissue samples were rinsed thoroughly with PBS before each extraction and cut with single edge double sided razor blades. The sea cucumber dermis was diced into small pieces. Tendon tissue was treated differently. For patellar tendons, a few shallow cuts were made longitudinally along the tendons. The cuts were intended to be deep enough to disrupt the endotenon, but not deep enough to cut through the whole tissue. Rat tail tendons fascicles were transversely cut into < 2 mm long segments with scissors.

### Trypsin extraction medium treatment

Samples were shaken using a rapid wrist motion in 3 ml trypsin extraction medium for ~5 min resulting in swollen tissue. Sea cucumber dermis pieces started out having well defined shapes with smooth surfaces. Upon agitation, the tissue swelled slightly and the solution became cloudy. No further changes in the appearance of the surfaces of the sea cucumber dermis tissue occurred. Tendon tissue behaved differently. The tissue began with a glossy sheen. After a few minutes of agitation, surface fibrillations became evident. These regions had a matt sheen and began as localized eruptions. With further agitation, most of the tissue volume converted into a “fluffy” matt finish with only a small central portion of tissue still looking shiny. This swelling did not occur when tissues were treated using the extraction medium without trypsin. In addition, swelling did not occur, even with trypsin exposure, if the shallow cuts were not first made to disrupt the endotenon. It was necessary not to over-agitate the tissue as this caused entanglement of fibrils clearly visible under dark-field imaging. This reduced the yield. After swelling stopped, the tissue was removed from solution with tweezers, rinsed with 5 ml extraction medium, followed by 10 ml PBS using a 1 ml pipette, and stored in 3 ml PBS at 4 ^°^C for < 7 d prior to fibril extraction.

### Fibril extraction

One piece of the swollen tissue was immersed in 2.5 ml PBS on a watch glass. Four points on the tissue were pinned with two pairs of sharp tweezers held slightly open. The tweezers were pulled in opposite directions at approximately 20 mm/s. This process was repeated until the tissue was pulled into a flat sheet ~2 cm on a side. The tissue was then stretched until it broke into two parts. The parts were subsequently aligned in parallel and the stretching process repeated. After ~10 times, the tissue became compacted presumably due to entanglement of the fibrils. Visible tissue pieces were removed and the remaining solution, which contained free fibrils, collected.

### Water-based extraction

Fibrils from the trypsin extraction method were compared against water-extracted fibrils from sea cucumber dermis [27]. In brief, pieces of sea cucumber dermis ~10 mm × 2 mm × 1 mm were stirred overnight in extraction medium without trypsin, rinsed 4 times by soaking in deionized water for 15 min and stirred in deionized water overnight at 4^°^C. The resulting solution, containing concentrated collagen fibrils, was diluted ~1000:1 before use.

### Mechanical extraction

Trypsin extracted fibrils were also compared against previously published methods that used robust mechanical disruption [22,24,25]. For these experiments, rat patellar tendons and sea cucumber dermis were cut into < 2 mm × 2 mm pieces with a surgical blade. 4–6 of these pieces were immersed in 1.5 ml PBS, and homogenized (Omni Tip™ Homogenizing Kit, Omni International, Kennesaw, GA) repeatedly at 35000 rpm (30 s each repeat) until the solution became cloudy. The resulting solution contained free fibrils as verified by dark-field microscopy. The length distributions of rat and sea cucumber fibrils obtained using homogenization were compared with the trypsin-extracted and water-extracted fibrils.

### Optical microscopy of single fibrils in solution

Fibrils were prepared for imaging as previously described [11,20]. Briefly, a droplet of fibril containing solution was deposited on a glass slide placed under an optical microscope. Typical bright-field contrast allowed visualization of collagen fibrils only after the solvent evaporated. To visualize collagen fibrils directly in aqueous solution, dark-field contrast was used using a 10 × objective lens [29]. Although the diameter of the fibrils were smaller than the diffraction limit, since the fibrils were separated far apart from each other, individual fibrils could still be visualized and identified clearly. Since most fibrils were relatively straight, end to end fibril distances were measured and used as an indication of fibril length.

### Scanning electron microscope imaging of single collagen fibrils

**Figure 2.**
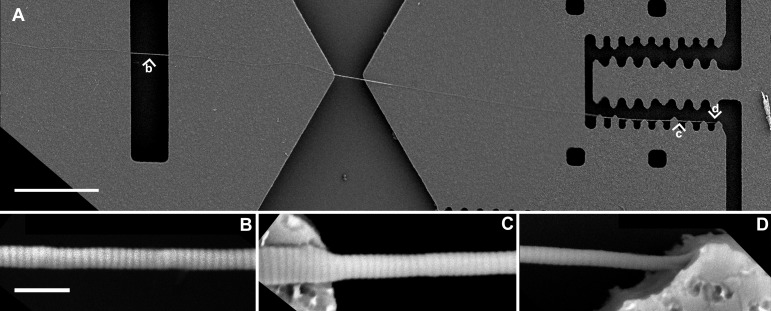
Scanning electron microscopy images of an extracted single collagen fibril from rat patellar tendon (long thin strand running left-right) atop a mechanical test device. Scale bar above represents 30 μm; scale bar below represent 500 nm. Master image at the top shows the entire fibril with length > 300 μm (**A**); the images below show sections on the same collagen fibril at regions labeled with small arrow heads b, c and d, corresponding to B, C and D: fibril diameter ~200 nm imaged over viewing window (**B**); diameter ~200 nm over gap widens to ~300 nm on the silicon surface (**C**); and diameter tapering at end of fibril consistent with an intact fibril terminus (**D**).

After a fibril was visualized in solution, it was grabbed using a pulled glass micropipette with a < 1 μm diameter tip attached to a hydraulic micromanipulator (model MM0-203, Narishige International, USA). The fibril was attracted to the micropipette presumably due to electrostatic forces. Once attached to the micropipette, the fibril was removed from solution and deposited on the surface of a silicon based testing device (**Fig. 2A**). 10 nm of palladium was then sputter coated onto the silicon devices (Desk IV, Denton Vacuum, Moorestown, NJ). The coated fibrils were visualized using a scanning electron microscope (Hitachi S4500). To determine fibril diameters, a 20 μm portion of each fibril was collected at 50 k × magnification. The pixel size in these images was 2.1 nm (corresponding to ~2 collagen molecule diameters). Using Matlab, 3096 cross sections were measured across each image. Reported values in the Results sections are means of these cross sections.

### Electrophoresis of collagen samples

Two tissue sources were investigated to see if trypsin treatment resulted in changes at the molecular level as assessed by SDS polyacrylamide gel electrophoresis (SDS-PAGE): rat tail tendon and bovine skin. Rat tendon fascicles were dissected from two entire tails and cut into ~1 cm sections. Half of the fascicles were treated with trypsin extraction medium until the tissue swelled and then rinsed using PBS. The other half was rinsed with PBS without trypsin treatment. Both samples were washed in PBS for 5 min, soaked in acetone for 5 min, and soaked in 70% 2-propanol for another 5 min. Samples were then stirred in 100 ml 0.02 N acetic acid for 4 d at 4^°^C until homogeneous viscous solutions formed. These solutions were centrifuged at 28000 g for 2 h at 4^°^C. Two milliliter supernatant of each sample was collected and diluted with 8 ml 0.02 N acetic acid.

Reconstituted fibrils were fabricated from bovine skin collagen monomer (Purecol) solution. Two sets of collagen fibril gels were synthesized by adding 0.5 ml Purecol solution to 10 ml PBS and incubating at 37^°^C overnight. This produced two loose gels consisting of ~75 nm diameter D-banded fibrils [13]. One sample was treated with > 2000 BAEE unit/ml trypsin for >10 min. Each sample was then collected using a 1 ml pipette, and dissolved in 3 ml 0.02 M acetic acid. It was assumed that if the trypsin cleaved the collagen, fragments would be trapped within the remaining gel and would show up as low molecular weight bands in the electrophoresis measurement. Samples were analyzed using SDS-PAGE with a 20% acrylamide gel under reducing conditions and stained with Coomassie brilliant blue overnight for protein visualization.

**Figure 3.**
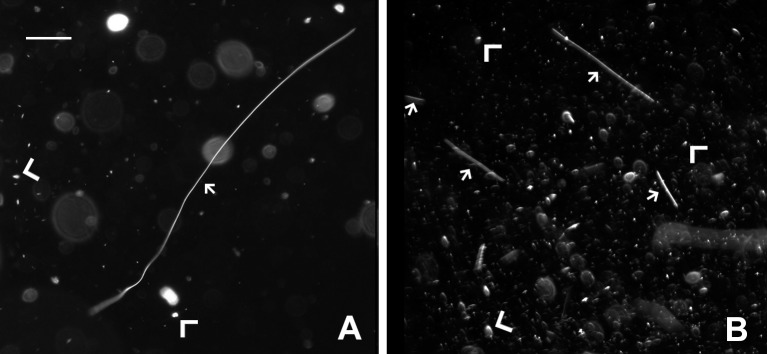
**Dark-field microscopy images of rat patellar tendon collagen fibrils in solutions using the new method presented in this paper (A) and tissue homogenization (B) (scale bar represents 100 μm).** Small arrows point out some suspended collagen fibrils. Large arrow heads point out non-fibraller tissue components. The fibril extracted without using vigorous mechanical disruption (**A**) is > 1000 μm. The fibrils extracted using tissue homogenization (**B**) vary in length from 30 μm to 400 μm.

## RESULTS

We first applied the least disruptive method, water extraction, to pieces of tendon tissue. While water-extraction produced fibrils from sea cucumber dermis [27], it produced no fibrils in solution using either rat tail or patellar tendon. Extraction using trypsin and using tissue homogenization produced fibrils from all tissue types. Therefore, for sea cucumber samples, we compared among all three methods, but for rat tendon samples, we could only compare between trypsin extraction and homogenization. In addition, because of their extreme aspect ratio and toughness of their fascicular sheath, rat tail tendon fascicles became entangled in the rotor. Thus, homogenized rat tail tendons were dismissed for this comparison. Finally, we performed length analyses on all fibrils because these tests only required optical microscopy examination. Diameter analyses were not performed on the homogenized fibrils because we used a technique requiring manipulation of individual fibrils out of solution for electron microscopy. The homogenized fibrils proved too short for this manipulation.

**Figure 4.**
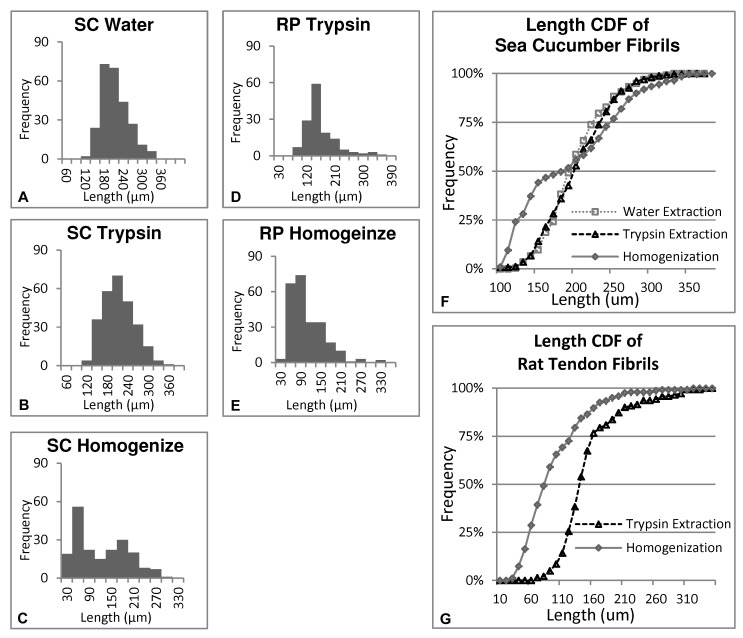
**Length distribution. A.** Sea cucumber dermis (SC) collagen fibrils extracted by deionized water. **B.** Sea cucumber dermis collagen fibrils extracted by the trypsin extraction medium. **C.** Sea cucumber dermis collagen fibrils extracted by tissue homogenization. **D.** Rat patellar tendon (RP) collagen fibrils extracted by the trypsin extraction medium. **E.** Rat patellar tendon collagen fibrils extracted by tissue homogenization. **F.** Cumulative (length) distribution frequency (CDF) of sea cucumber dermis collagen fibrils extracted by deionized water, trypsin and homogenization. **G.** Rat tendon collagen fibrils extracted by trypsin and homogenization.

### Fibril length

Fibril solutions were analyzed using dark-field microscopy (**Fig. 3**). While fibril widths are below the diffraction limit of visible light, dark-field imaging provides sufficient contrast for the fibrils to appear clearly as high aspect ratio bright strands showing fibril morphology at the micro-level (small arrows in **Fig. 3**). The lengths of fibrils extracted from rat patellar tendons with the trypsin-based method varied from 120 μm to 160 μm, with maximum lengths greater than 1000 μm like the fibril indicated with a small arrow in **Figure 3A**. In contrast, the lengths of fibrils obtained using tissue homogenization varied from 50 μm to 120 μm (small arrows in **Fig. 3B**). The optical fields of view also showed non-fibrillar objects (indicated with large arrow heads in **Fig. 3**). These objects did not impede our ability to isolate single fibrils from solution.

To compare different extraction methods quantitatively, lengths of > 140 fibrils from each sample were measured with dark-field microscopy (**Fig. 4**). For sea cucumber dermis, the mean length of water-extracted fibrils was 198 ± 43 μm (n = 257), trypsin-extracted fibrils was 200 ± 45 μm (n = 270), and homogenization extracted fibrils was 109 ± 69 μm (n = 200). For rat patellar tendon, trypsin-extracted fibrils had mean length of 150 ± 50 μm (n = 142), and homogenization extracted fibrils had mean length of 95 ± 50 μm (n = 245). The length distributions for the water and trypsin extracted fibrils (**Fig. 4A, 4B** and **4D**) appear unimodal with single peaks near their means and little evidence of asymmetry in the distributions. The length distributions for homogenization extracted fibrils (**Fig. 4C** and **4E**) are clearly less symmetric. The length distribution of the sea cucumber fibrils (**Fig. 4C**) shows two peaks, one near 60 μm and one near 180 μm. The 180 μm peak appears near the center of the belly of the distributions for the water and trypsin extracted sea cucumber fibrils. The rat patellar tendon fibrils extracted by homogenization (**Fig. 4E**) do not show two distinguishable peaks in their length distribution. However, the main peak is clearly at shorter lengths than the trypsin-extracted fibrils from this tissue (**Fig. 4D**). In addition, there is a fairly clear asymmetry to the homogenized length distribution for rat patellar tendons with a long fibril length shoulder evident between 120 μm and 210 μm. Wilcoxon Rank-sum Tests were conducted between different sample populations to determine statistical significance. The test showed no significant difference between the length of water-extracted and trypsin-extracted sea cucumber fibrils (*P* = 0.51). Conversely, the homogenization extracted sea cucumber fibrils were significantly shorter than both water (*P* < 0.01) and trypsin extracted sea cucumber fibrils (*P* < 0.01). The mean length of homogenization extracted rat patellar tendon fibrils was significantly shorter than the trypsin-extracted rat patellar tendon fibrils (*P* < 0.01).

**Figure 5.**
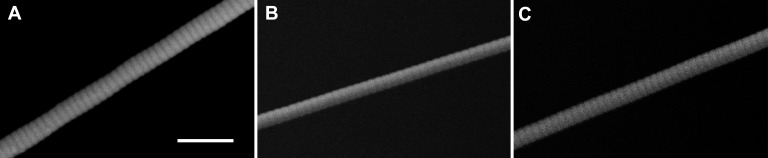
**Scanning electron microscopy images of collagen fibrils showing that D-banding is not extinguished by trypsin treatment. A.** An extracted rat patellar tendon collagen fibril obtained with the trypsin extraction method. **B.** An extracted sea cucumber dermis collagen fibril obtained with the trypsin-based extraction method. **C.** An extracted sea cucumber dermis collagen fibril obtained with the water extraction method. All three collagen fibril samples show clear D-banding patterns on the surface of the fibril, consistent with undamaged surface structure at the nano level. Scale bar is 500 nm.

### D-banding

Morphological fibril damage at smaller length scales was investigated using scanning electron microscopy. Qualitative assessment of D-banding patterns was possible. If there were extensive damage at this level, it was expected that D-banding would be disrupted and that variations in fibril diameter would increase compared to undamaged fibrils. Electron microscopy images showed that trypsin-extracted patellar tendon fibrils showed a clear D-banding pattern on the fibril surface (**Fig. 5A**). Sea cucumber fibrils were used to make a direct comparison between the trypsin extraction (**Fig. 5B**) and water extraction (**Fig. 5C**). D-banding was clearly evident in both cases. The presence of banding in the electron microscopy images is consistent with a lack of surface disruption of the fibrils at sub-micron length scales.

### Fibril diameter

Quantitative comparison of the variation in diameter along the fibril axis was made using 7 water-extracted and 7 trypsin-extracted sea cucumber fibrils. Since the individual fibrils examined had different diameters, a comparison metric that normalized for this was needed. We chose the standard deviation of the fibril diameter divided by the average fibril diameter. Each of these numbers was computed using over 3000 cross-sections taken over a 20 µm stretch of fibril. The 20 μm region was chosen at random from a section of fibril > 50 μm from its terminus, because we have observed that mammalian collagen fibrils usually have relative uniform diameters after the 50 μm form their tapered ends, and with appropriate magnifications, each electron micrograph showed at least 20 μm fibril. **Figure 2** shows an example of the type of fibril image used for this analysis. The sections of the fibril in **Figure 2B** and **2C** that are suspended over openings in the silicon device show a uniform ~200 nm diameter. It is important to make these measurements on suspended portions of the fibril since substantial spreading occurs on the silicon substrate [11] as can be seen near the left side in **Figure 2C**. For the water-extracted control group, the standard deviation of the diameters was 8% ± 3% of the mean diameter. For the trypsin treated group, the standard deviation of the diameters was 7% ± 2% of the mean diameter. A Student’s *t*-test showed no significant difference between the trypsin treated and trypsin untreated sample populations (*P* = 0.69).

Fibril termini provide additional information regarding morphological damage at this length scale. The fibril in **Figure 2D** shows a tapered end indicative of the natural fibril end as opposed to the more blunt terminations expected from fibrils fractured due to treatments like tissue homogenization. Finding the ends of the fibrils in the scanning electron microscope was difficult, so this was not attempted for all fibrils on which we took diameter measurements.

### Molecular scale comparison

**Figure 6.**
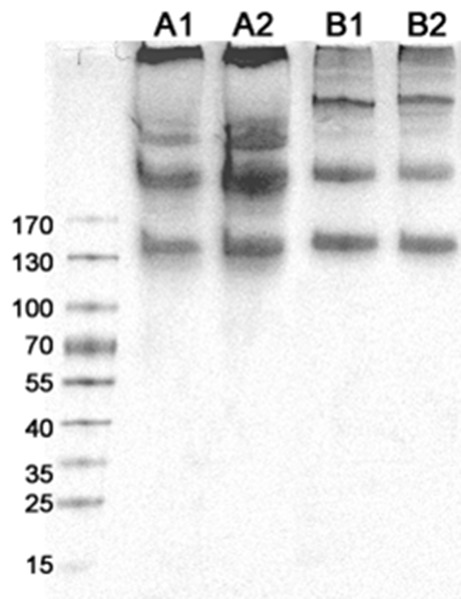
**SDS-PAGE of collagen samples stained with Coomassie brilliant blue.** Sample set A was prepared from the rat tail tendon collagen stock solution diluted by 1:4, and sample set B was prepared from synthetic collagen gel made from commerical bovine skin collagen solution (Purecol). All samples showed three band marks of molecular weights above 130 kDa. Lane A1: Rat tail tendon collagen molecules without trypsin treatment; A2: Rat tail tendon collagen molecules with trypsin treatment at the fibril level; B1: Bovine skin collagen without trypsin treatment; B2: Bovine skin collagen with trypsin treatment. No significant difference was observed within each sample set. No evidence of discrete digestion products between 15 and 130 kDa in any lane suggesting that trypsin does not cleave either rat tail tendon collagen or bovine skin collagen.

Molecular scale damage was evaluated using electrophoresis to compare molecular weight distributions of samples with and without trypsin treatment (**Fig. 6**). Trypsin treated collagen molecules from rat tendon (lane A2) and reconstituted bovine skin collagen fibrils (lane B2) are shown beside their control lanes of non-trypsin treated rat tendon (lane A1) and reconstituted bovine skin collagen fibrils (lane B1) respectively. Only three bands are seen in each lane. The centers of the bands in lanes A1 and A2 are nearly identical as are the centers of the bands in lane B1 and B2.

## DISCUSSION

In general, solutions produced using the trypsin-based method had more uniform longer fibrils than tissue homogenized fibril solution. Trypsin is a commonly used proteinase in biochemical studies. It digests many protein molecules, but does not cause significant damage to intact collagen molecules [30-32]. Insensitivity to trypsin digestion is a standard characteristic of intact collagen molecules according to ASTM standard F2212 [33]. Thus, it is not surprising that no measurable damage to the type I collagen fibrils due to trypsin exposure was found.

It is natural to speculate about why trypsin works in the separation of collagen fibrils from their neighbors. We stumbled on this choice of enzyme after trying less physiologically specific treatments such as altering the pH and ionic strength of the extraction medium to increase charge on the surface of adjacent fibrils and to reduce Debye screening acting to shield the charges. When these strategies failed, we tried hyaluronidase, a carbohydrate enzyme that could digest some of the sugars existing in the ground substance between fibrils [34]. This did not work either. We then sought a proteinase that would not digest collagen. Being aware of the ASTM F2212 standard, we decided to try trypsin. While it is well-known that many noncollagenous proteins exist in the ground substance [35], it is also well-accepted that we do not know how these molecules are arranged [36]. Thus, we do not wish to speculate at this time on the precise mechanism by which trypsin allows the liberation of collagen fibrils from their neighbors in tendon tissue. We only conclude that the procedure presented works, and leave mechanistic studies to future work.

Dark-field microscopy provided morphological evidence that collagen fibrils extracted with the trypsin-based method were significantly longer than fibrils extracted by tissue homogenization. In addition, trypsin-extracted fibrils showed only one population as evidenced by the unimodal distributions in **Figure 4B** and **4D** homogenization extracted sea cucumber showed a bimodal distribution, with a minor mode of longer fibrils likely corresponding to a sub-population of water-extracted fibrils and a major mode of shorter fibrils (**Fig. 4C**). This is consistent with a large portion of damaged fibrils coexisting with another fraction of undamaged fibrils. Homogenization extracted rat patellar tendon fibrils showed an asymmetric distribution of predominantly shorter fibrils (**Fig. 4E**) compared to the trypsin-extracted rat patellar tendon fibrils (**Fig. 4D**). Shortening of fibrils by tissue homogenization is likely caused by fracture failure. A concern is, even if a relatively long fibril were extracted from a solution prepared using tissue homogenization, there is no guarantee that sub-fracture damage is not present in that fibril. Rigorously speaking, there is no guarantee that such damage is not also present in fibrils prepared using the less mechanically disruptive method presented in this paper. However, since no evidence was found that the new method is any more damaging than soaking in EDTA and water, it seems less likely that undetectable sub-rupture damage exists in the trypsin-extracted fibrils compared with fibrils exposed to tissue homogenization.

Scanning electron microscopy showed the trypsin-extracted sea cucumber fibrils had similar diameter variation along the length of the fibrils compared with water-extracted fibrils. Previously published scanning electron microscopy images of sea cucumber fibrils showed uniform diameter near the middle region of the fibril [12,20]. However, TEM images in earlier publications [18,19] show the diameter changes by about 1 nm for every 1 μm of travel along the fibril axis. Analyzing a 20 μm stretch of a fibril with mean diameter of 100 μm, this corresponds to a change in the computation we performed (standard deviation/mean diameter) of ~7%. The diameter variation results of water-extracted fibrils (8% ± 3%) and trypsin-extracted fibrils (7% ± 2%) fall clearly within the range one might expect for fibrils extracted from sea cucumber dermis. Effects the trypsin method had on fibril diameter were small compared to this expected natural diameter variation. This diameter variation analysis in addition to clear D-banding in both trypsin and water extracted sea cucumber fibrils, lead us to conclude that there was no evidence of enzymatic cleavage or other chemical damage leading to morphological changes on the surface of the fibrils. This supports that trypsin-extraction does not cause more morphological damage than the water-extracted collagen fibrils.

To investigate the potential molecular level degradation of collagen caused by trypsin digestion, electrophoresis was used to compare the molecular weight distribution of collagen samples before and after trypsin treatment. SDS-PAGE results (**Fig. 6**) showed no evidence that trypsin treatment of collagen fibrils caused chain scission. We expected to find three bands corresponding to single, double and triple alpha helices in undamaged samples. Damaged samples were expected to show lower molecular weight bands corresponding to partially digested molecules. **Figure 6** clearly shows only three relatively high molecular weight bands from each sample. Fibrils reconstituted from bovine skin collagen showed no difference in molecular weight distribution with (**Fig. 6**, lane B2) and without (**Fig. 6**, lane B1) trypsin treatment. Additionally, fibrils extracted from tendon showed similar molecular weight distributions with (**Fig. 6**, lane A2) and without (**Fig. 6**, lane A3) trypsin treatment. These SDS-PAGE results show that trypsin extraction does not cause chain scission of collagen molecules that could potentially change physical properties at the sub-fibrillar level. Strong conclusions regarding molecular level changes such as cleavage of telopeptides and/or effects on crosslinking due to trypsin treatment will require more careful sample preparation techniques than the ones used in this study.

It is worth considering further the effect trypsin might have on crosslinks within a fibril in addition to direct effects on the collagen molecule itself. Disruption of crosslinks would likely change the physical properties of the fibril. However, trypsin has a molecular weight in excess of 23 kDa. A molecule of this size could not fit between the collagen molecules in an assembled fibril. Thus, trypsin might disrupt crosslinks on the surface of the fibril. However, attractive forces that originally drove assembly of the fibril would likely prevent surface molecules from drifting away from their starting positions. This would prevent trypsin from gaining access to the fibril interior to cause further disruption.

The results of light microscopy, electron microscopy and electrophoresis all indicated that trypsin causes less fibril damage than homogenization, and that trypsin exposure followed by gentle manual tensioning is useful in extracting collagen fibrils from mammalian tendons without causing significant morphological damage to the fibrils. Collagen fibrils extracted using our method can be transferred onto a microscale test machine for further studies (**Fig. 2**). Since tendinous tissues have similar biological structure in different mammalian species [37], the trypsin extraction method should also work on other mammalian tendinous tissues including human tendons in future studies. The method will also help determine biomaterial design targets for actual collagen fibril length based on measures of natural fibrils.

In conclusion, the trypsin-based collagen fibril extraction method introduced above was verified to be effective on both positional (tail) and load bearing (patellar) tendons. Comparisons at the molecular and whole fibril level between trypsin-extracted and water-extracted samples showed no differences. Comparisons between trypsin-extracted and tissue homogenized samples showed significantly shorter fibrils from the homogenized group. Compared with fibril extraction by vigorous disruption [22-26], the method presented in this paper applies less physical damage to the samples and allows the fibrils to remain hydrated through the entire process preventing changes caused by dehydration. These benefits will help obtain more morphologically intact collagen fibrils. Finally, the trypsin-based method was verified not to decrease the length of sea cucumber dermis fibrils, not to extinguish D-banding in rat patellar or sea cucumber dermis fibrils, and not to change the molecular weight of collagen molecules from reconstituted purified calf skin dermis collagen gels or rat patellar tendons. Thus, the trypsin-extraction method can be used to compare the difference among collagen fibrils obtained from different sources while processing all the tissues with the identical procedure. This will eliminate concerns about differences in results due to processing effects.
